# Insurance Type and Withdrawal of Life-Sustaining Therapy in Critically Injured Trauma Patients

**DOI:** 10.1001/jamanetworkopen.2024.21711

**Published:** 2024-07-24

**Authors:** Graeme Hoit, Duminda N. Wijeysundera, Doulia M. Hamad, Aaron Nauth, Amit Atrey, Mansur Halai, Eric Walser, Anton Nikouline, Avery B. Nathens, Amir Khoshbin

**Affiliations:** 1 Department of Surgery, Division of Orthopaedic Surgery, University of Toronto, Toronto, Ontario, Canada; 2 Institute of Health Policy, Management and Evaluation, University of Toronto, Toronto, Ontario, Canada; 3 Department of Anesthesia, St Michael’s Hospital, Toronto, Ontario, Canada; 4 Department of Anesthesiology and Pain Medicine, University of Toronto, Toronto, Ontario, Canada; 5 Department of Surgery, Division of General Surgery, University of Toronto, Toronto, Ontario, Canada; 6 Division of Orthopaedic Surgery, St Michael’s Hospital, University of Toronto, Toronto, Ontario, Canada; 7 Instutitue of Medical Sciences, University of Toronto, Toronto, Ontario, Canada; 8 Department of Surgery, Western University, London, Ontario, Canada; 9 Division of Critical Care, Western University, London, Ontario, Canada; 10 Division of Emergency Medicine, Western University, London, Ontario, Canada; 11 American College of Surgeons, Chicago, Illinois

## Abstract

**Importance:**

Withdrawal of life-sustaining therapy (WLST) decisions for critically injured trauma patients are complicated and multifactorial, with potential for patients’ insurance status to affect decision-making.

**Objectives:**

To determine if patient insurance type (private insurance, Medicaid, and uninsured) is associated with time to WLST in critically injured adults cared for at US trauma centers.

**Design, Setting, and Participants:**

This retrospective registry-based cohort study included reported data from level I and level II trauma centers in the US that participated in the American College of Surgeons Trauma Quality Improvement Program (TQIP) registry. Participants included adult trauma patients who were injured between January 1, 2017, and December 31, 2020, and required an intensive care unit stay. Patients were excluded if they died on arrival or in the emergency department or had a preexisting do not resuscitate directive. Analyses were performed on December 12, 2023.

**Exposures:**

Insurance type (private insurance, Medicaid, uninsured).

**Main Outcomes and Measures:**

An adjusted time-to-event analysis for association between insurance status and time to WLST was performed, with analyses accounting for clustering by hospital.

**Results:**

This study included 307 731 patients, of whom 160 809 (52.3%) had private insurance, 88 233 (28.6%) had Medicaid, and 58 689 (19.1%) were uninsured. The mean (SD) age was 40.2 (14.1) years, 232 994 (75.7%) were male, 59 551 (19.4%) were African American or Black patients, and 201 012 (65.3%) were White patients. In total, 12 962 patients (4.2%) underwent WLST during their admission. Patients who are uninsured were significantly more likely to undergo earlier WLST compared with those with private insurance (HR, 1.54; 95% CI, 1.46-1.62) and Medicaid (HR, 1.47; 95% CI, 1.39-1.55). This finding was robust to sensitivity analysis excluding patients who died within 48 hours of presentation and after accounting for nonwithdrawal death as a competing risk.

**Conclusions and Relevance:**

In this cohort study of US adult trauma patients who were critically injured, patients who were uninsured underwent earlier WLST compared with those with private or Medicaid insurance. Based on our findings, patient’s ability to pay was may be associated with a shift in decision-making for WLST, suggesting the influence of socioeconomics on patient outcomes.

## Key Points

**Question:**

Is insurance type associated with time to withdrawal of life-sustaining therapy in adult trauma patients in the US?

**Findings:**

In this cohort study of 307 731 patients, patients who were uninsured were significantly more likely to undergo earlier withdrawal of life-sustaining therapy compared with those with private insurance and Medicaid.

**Meaning:**

These findings suggest that being uninsured is associated with a shift in decision-making toward earlier withdrawal of life-sustaining therapy, which could indicate that socioeconomic status informs end-of-life care for patients with critical injuries.

## Teaser

This cohort study examines patients’ insurance type and the decision to withdraw life-sustaining treatment in trauma patients who are critically injured using data from level I and level II trauma centers in the US.

## Introduction

Withdrawal of life-sustaining therapy (WLST) decisions are complicated and multifactorial for trauma patients who are critically injured. Ideally, WLST is an individualized shared decision made by health care clinicians and the patient’s substitute decision-makers (SDMs) in accordance with the patient’s previously outlined advanced directives and values, with integration of cultural competencies and illness prognosis.^1,2^ However, the nature of severe traumatic injury means that trauma patients are typically younger, less likely to have preexisting care directives, more likely to be estranged from their families and SDMs, and more likely to belong to marginalized social populations compared with the general critical care population.^3^ These factors complicate WLST decisions and may increase the likelihood of practitioner, caregiver, or institutional biases impacting decisions and timing.

In 1986, the Emergency Medical Treatment and Labor Act was enacted to ensure that all patients who are critically ill be provided optimal care regardless of financial means or insurance status.^4^ However, the decision to proceed with escalating care or heroic measures after initial stabilization of the injured patient may have significant financial implications for patients, their families, and institutions when a patient is uninsured. Over 70% of patients who are uninsured admitted for trauma are at risk of catastrophic health expenditures.^5^ Thus, in the absence of preexisting directives, the financial cost of deciding to continue or escalate treatment may factor into SDM decisions surrounding WLST.^6,7,8^

When patients are unable to pay for health care expenditures, the financial responsibility for the care provided may land on the institutions themselves. Trauma centers across the United States face a disproportionately high burden of providing care for patients who are uninsured. A 2013 study estimated an annual care cost of over $2.8 billion from uninsured trauma care.^9^ Accordingly, there may be disincentive from an institutional perspective in deciding to treat or escalate care for the patients who are uninsured or have Medicaid in comparison to patients who are privately insured.^10,11^

With this study, our primary objective was to determine if patient insurance type (private insurance, Medicaid and uninsured) is associated with time to WLST in critically injured adults cared for at US trauma centers. We hypothesized that patients who are uninsured would undergo earlier WLST compared with patients who are privately insured. Secondarily, we aimed to analyze other baseline factors that were associated with time to WLST.

## Methods

### Study Design, Data Source, and Research Ethics

We conducted a registry-based retrospective cohort study using data from the American College of Surgeons Trauma Quality Improvement Program (TQIP) over 4 years (2017 to 2020). TQIP is a registry of more than 700 participating trauma centers across the US. Patients with at least 1 severe injury (abbreviated injury score [AIS] of 3 or more in at least 1 body region) are included. Data quality is ensured through trained data abstractors collecting and validating data entry and interrater reliability external audits performed. This project was approved by the St Michael’s Hospital research ethics board. The need for patient informed consent was waived due to the deidentified nature of the data. This study followed the Strengthening the Reporting of Observational Studies in Epidemiology (STROBE) reporting guideline.^12^

### Population

Patients aged 18 to 64 years treated at level I and II trauma centers participating in TQIP between January 1, 2017, and December 31, 2020, were considered. January 2017 was selected as the starting point because this corresponds with the introduction of the WLST variables within the TQIP registry. Assembly of the study cohort is shown in the Figure. We included Medicaid, private insurance, and uninsured trauma patients who required admission to the intensive care unit. Patients with missing insurance status and those with Veterans Affairs insurance or Medicare (in patients younger than 65 years limited only to those receiving social security disability and those with end stage kidney disease or amyotropic lateral sclerosis) were not considered in our study. We also excluded those with preexisting do not resuscitate (DNR) orders, those with missing withdrawal status, and those with missing or other insurance status.

**Figure.**
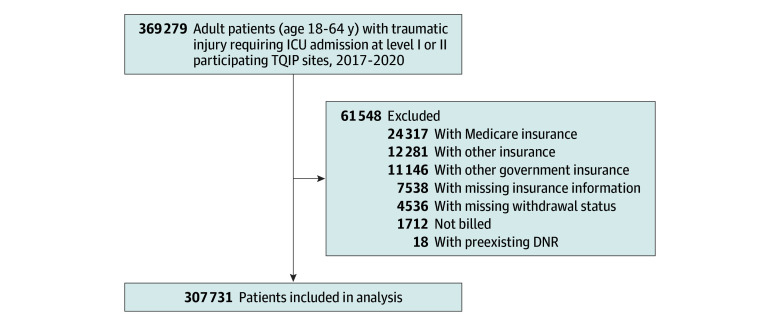
Assembly of the Patient Cohort DNR indicates do not resuscitate; ICU, intensive care unit; TQIP, American College of Surgeons Trauma Quality Improvement Program.

### Study Outcome and Exposure

The primary outcome of this study was time to WLST, as defined by days after admission. WLST is defined by TQIP as a documented “decision to remove or withhold further life supporting intervention.”^40^ According to their definition, life supporting interventions are limited to ventilator support (with or without extubation), kidney replacement therapy, medications to support blood pressure or cardiac function, and surgical, interventional, or radiological procedures. Patient insurance status was the exposure variable of interest.

### Other Potential Factors Affecting WLST

We considered a broad range of patient, treatment, and clinician factors that have previously been shown to or could be thought to affect insurance status or WLST for inclusion in our statistical modeling a priori. Patient age, sex, race, ethnicity, comorbidities, injury mechanism, presenting vitals, presenting GCS, max AIS score, and whether they underwent interfacility transfer have all previously been found to affect mortality, care decisions, and outcomes in trauma patients.^3,13,14,15^ Patient race and ethnicity are recorded in TQIP as self-reported by either the patient or a family member in accordance with the classifications in the US Census Bureau. Workplace injury, injury intent, hospital size, teaching status, and profit status were included because we felt these variables may also secondarily affect family and clinician treatment decisions. Comorbidities were included as a composite score using the trauma comorbidity index to account for the additive effect of multiple comorbidities in worsening prognosis.^16^

### Statistical Analysis

Descriptive statistics were calculated and presented as mean (SD) or median (IQR) for continuous variables where appropriate and relative frequencies with percentages for categorical variables. Standardized mean differences (SMDs) were used to compare characteristics between patients of different insurance types, with absolute differences greater than 10% indicating meaningful differences.^17^

We calculated adjusted hazard ratios (HRs) between insurance type and time to withdrawal of care using a shared frailty model (also known as mixed-effects survival model) using lognormal distribution with random effects and clustering by facility ID to account for institutional differences.^18^ Transformations of continuous variables were explored to best fit, and age was represented as a restricted cubic spline with 3 knots at the 10th, 50th, and 90th percentile. Relevant interaction terms were explored for significance (coma with insurance type, race with insurance type, and hospital profit status with insurance type), though none were left in the final model as they were either not statistically significant (race with insurance type, and hospital profit status with insurance type) or not felt to aid in explaining the data and results (coma with insurance type). Missing data were excluded from analyses.

We planned a priori sensitivity analyses. The first was excluding all patients who died within 48 hours of presentation to account for survivor treatment assignment bias, which has previously been documented for insurance status in trauma patients as hospitals are less likely to obtain insurance coverage for trauma patients uninsured who die early.^14,19^ The threshold of 48 hours was chosen because prior work investigating this phenomenon determined almost all patients who would ultimately be insured at discharged were insured by day 3.^14^ The second was to formally account for the competing risk of death before WLST using cause specific and subdistribution (Fine and Gray) hazard models.

All analyses were performed using SAS version 9.4 (SAS Institute). Analyses were performed on December 12, 2023. Statistical significance was set at *P* < .05, and all tests were 2-sided.

## Results

This study included 307 731 patients, of whom 160 809 (52.3%) had private insurance, 88 233 (28.6%) had Medicaid, and 58 689 (19.1%) were uninsured. The mean (SD) age was 40.2 (14.1) years, 232 994 (75.7%) were male, 59 551 (19.4%) were African American or Black patients, and 201 012 (65.3%) were White patients (Table 1).

**Table 1.** Cohort Characteristics by Insurance Type

Characteristic	Patients, No. (%)				SMD
	All patients	Medicaid	Private insurance	Self-pay	
**Patient characteristics**					
Total No.	307 731	88 233 (28.6)	160 809 (52.3)	58 689 (19.1)	NA
Age					
Mean (SD), y	40.2 (14.1)	40.0 (13.7)	41.7 (14.6)	37.8 (12.9)	0.19
Missing	NA	NA	NA	NA	
Sex					
Male	232 994 (75.7)	66 875 (75.8)	117 673 (73.2)	48 446 (82.6)	0.15
Female	74 668 (24.3)	21 342 (24.2)	43 105 (26.8)	10 221 (17.4)	
Missing	69 (0.02)	16 (0.02)	31 (0.02)	22 (0.04)	
Race					
African American or Black	59 551 (19.4)	23 081 (26.2)	20 802 (12.9)	15 668 (26.7)	0.32
American Indian or Native Alaskan	9050 (2.9)	3513 (4.0)	4444 (2.8)	1093 (1.9)	
Asian	29 027 (9.4)	9285 (10.5)	12 585 (12.2)	7157 (12.2)	
Native Hawaiian or Pacific Islander	1011 (0.3)	382 (0.4)	502 (0.3)	127 (0.2)	
White	201 012 (65.3)	49 512 (56.1)	118 596 (73.8)	39 904 (56.1)	
Other^a^	8080 (2.6)	2460 (2.8)	3880 (2.4)	1740 (3.0)	
Ethnicity					
Hispanic	44 875 (14.6)	15 289 (17.3)	18 093 (11.3)	11 493 (19.6)	0.16
Not Hispanic	249 362 (81.0)	68 934 (78.1)	136 072 (84.6)	44 356 (14.4)	
Missing	12 494 (4.4)	4010 (4.5)	6644 (4.1)	2840 (4.8)	
Comorbid conditions					
ADHD	4748 (1.5)	1417 (1.6)	2785 (1.7)	546 (0.9)	0.05
Alcohol use disorder	33 768 (11.0)	13 501 (15.3)	13 794 (8.6)	6473 (11.0)	0.14
Angina	158 (0.05)	48 (0.1)	104 (0.1)	6 (0.0)	0.02
Anticoagulant	7493 (2.4)	2010 (2.3)	4920 (3.1)	563 (1.0)	0.10
Bleeding disorder	2253 (0.7)	901 (1.0)	1140 (0.7)	212 (0.4)	0.05
Active chemotherapy	563 (0.2)	137 (0.2)	387 (0.2)	39 (0.1)	0.03
CHF	3005 (1.0)	1134 (1.3)	1602 (1.0)	269 (0.5)	0.06
Cirrhosis	4317 (1.4)	1967 (2.2)	1722 (1.1)	628 (1.1)	0.06
COPD	9178 (3.0)	3759 (4.3)	4405 (2.7)	1014 (1.7)	0.10
Congenital	1212 (0.4)	408 (0.5)	672 (0.4)	132 (0.2)	0.03
CVA	2565 (0.8)	1082 (1.2)	1227 (0.8)	256 (0.4)	0.06
Dementia	585 (0.2)	247 (0.3)	291 (0.2)	47 (0.1)	0.03
Diabetes	22 754 (7.4)	6411 (7.3)	13 754 (8.6)	2589 (4.4)	0.11
Disseminated cancer	735 (0.2)	201 (0.2)	477 (0.3)	57 (0.1)	0.03
Dependent functional status	4239 (1.4)	1912 (2.2)	2008 (1.3)	319 (0.5)	0.10
Hypertension	53 627 (17.4)	14 770 (16.7)	32 481 (20.2)	6376 (10.9)	0.17
Myocardial infarction	1109 (0.4)	344 (0.4)	641 (0.4)	124 (0.2)	0.02
Peripheral arterial disease	649 (0.2)	241 (0.3)	351 (0.2)	57 (0.1)	0.03
Mental health or personality disorder	32 524 (10.6)	12 800 (14.5)	15 670 (9.7)	4054 (6.9)	0.17
Kidney failure	1617 (0.5)	631 (0.7)	864 (0.5)	122 (0.2)	0.05
Smoking	89 255 (29)	31 264 (35.4)	38 531 (24.0)	19 470 (33.2)	0.17
Steroids	1239 (0.4)	323 (0.4)	826 (0.5)	90 (0.2)	0.17
Substance abuse disorder	37 979 (12.3)	17 014 (19.4)	13 541 (0.8)	7334 (12.5)	0.21
TCI					
Mean (SD)	−0.19 (0.50)	−0.29 (0.58)	−0.13 (0.45)	−0.23 (0.47)	0.21
Missing	NA	NA	NA	NA	NA
**Injury characteristics**					
ISS Score					
Mean (SD)	20.8 (10.3)	20.6 (10.4)	20.9 (10.2)	20.6 (10.4)	0.02
Missing					
AIS >3, No. (%)					
Head	74 973 (24.4)	22 2347 (25.3)	37 966 (23.6)	14 660 (25.0)	0.03
Face	429 (0.1)	130 (0.2)	214 (0.1)	85 (0.1)	0
Neck	1643 (0.5)	590 (0.7)	739 (0.5)	314 (0.5)	0.02
Chest	27 151 (8.8)	7823 (8.9)	14 055 (8.7)	5273 (9.0)	0.01
Abdomen	28 108 (9.1)	8352 (9.5)	13 857 (8.6)	5899 (10.1)	0.03
Spine	14 811 (4.8)	4716 (5.3)	7859 (4.9)	2236 (3.8)	0.05
UE	443 (0.1)	116 (0.1)	262 (0.2)	65 (0.1)	0.01
LE	11 366 (3.7)	3118 (3.5)	6395 (4.0)	1853 (3.2)	0.03
Coma, presenting GCS ≤8	78 013 (25.4)	23 437 (26.6)	38 352 (23.9)	16 224 (27.6)	0.06
Injury mechanism					
Fall	68 264 (22.2)	20 760 (23.5)	36 267 (22.6)	11 237 (19.2)	0.42
Firearm	36 823 (12.0)	15 831 (17.9)	9304 (5.8)	11 688 (19.9)	NA
MVC	107 013 (34.7)	22 493 (25.5)	66 994 (41.7)	17 526 (29.9)	NA
Motorcycle	30 300 (9.9)	5717 (6.5)	19 764 (12.3)	4819 (8.2)	NA
Pedestrian	28 946 (9.4)	9451 (10.7)	14 294 (8.9)	5201 (8.9)	NA
Stab	11 229 (3.7)	5156 (5.8)	3090 (1.9)	2983 (5.1)	NA
Struck	19 428 (6.3)	7262 (8.2)	7881 (4.9)	4285 (7.3)	NA
Other	3246 (1.0)	674 (0.8)	2036 (1.3)	536 (0.9)	NA
Missing	NA	NA	NA	NA	NA
Intent					
Assault	48 699 (15.8)	23 234 (26.3)	11 272 (7.0)	14 193 (24.2)	0.39
Other	887 (0.3)	372 (0.4)	241 (0.2)	274 (0.5)	
Self-inflicted	8678 (2.8)	2932 (3.3)	3452 (2.2)	2294 (3.9)	
Undetermined	2411 (0.8)	924 (1.1)	675 (0.4)	812 (1.4)	
Unintentional	247 056 (80.3)	60 771 (68.9)	145 169 (90.3)	41 116 (70.1)	
Missing	0	NA	NA	NA	
Work-related					
Yes	16 144 (5.3)	1348 (1.5)	12 638 (7.9)	2158 (3.7)	0.21
No	289 036 (93.9)	86 167 (97.7)	146 778 (91.3)	56 091 (95.6)	
Missing	2551 (0.8)	718 (0.8)	1393 (0.9)	440 (0.8)	
**Treatment characteristics**					
Hospital teaching status					
Teaching	160 438 (52.1)	48 310 (54.8)	79 361 (49.4)	32 767 (55.8)	0.09
Non-teaching	144 657 (47.0)	39 299 (44.5)	80 214 (49.9)	25 177 (42.9)	
Missing	2636 (0.9)	657 (0.7)	1235 (0.8)	745 (1.3)	
Hospital financial type					
For profit	35 661 (11.6)	7601 (8.6)	18 129 (11.3)	9931 (16.9)	0.04
Non-profit	272 070 (88.4)	80 632 (91.4)	142 680 (88.7)	48 758 (83.1)	
Missing	0	NA	NA	NA	
Bed size					
≤200	89 143 (29.0)	24 522 (27.8)	47 373 (29.5)	17 248 (29.4)	0.13
201-400	61 020 (19.8)	18 459 (20.9)	33 007 (20.6)	9484 (16.2)	
401-600	70 820 (23.1)	24 522 (27.8)	34 668 (21.6)	13 539 (23.1)	
>600	86 748 (28.2)	22 639 (25.7)	45 691 (28.4)	18 418 (31.4)	
Missing	NA	NA	NA	NA	

Abbreviations: ADHD, attention deficit/hyperactivity disorder; AIS, Abbreviated Injury Score; CHF, congestive heart failure; COPD, chronic obstructive pulmonary disorder; CVA, cerebrovascular accident; GCS, Glasgow Coma Scale; ISS, Injury Severity Score; LE, lower extremity; MVC, motor vehicle collision; NA, not available; SMD, standardized mean difference; TCI, Trauma Comorbidity Index; UE, upper extremity.

^a^ Other race is inclusive of patients whose race was unknown, not reported, or missing.

Compared with patients with private insurance, patients who were uninsured were younger; more likely to be male and from minoritized racial and ethnic groups; had more substance abuse; had lower rates of known chronic illness; and were more likely to be victims of assault (Table 1). In most data categories, patients with Medicaid were most similar to patients who were uninsured.

In total, 12 962 patients (4.2%) underwent WLST during their admission, with a higher proportion in population of patients without insurance (2949 [5.0%]) compared with Medicaid (3713 [4.2%]) and private insurance (6300 [3.9%]; SMD, 0.04). The mean time to WLST was 7.8 (10.4) days in the cohort, with shorter mean WLST in the uninsured group (6.5 [9.83] days), followed by private insurance (7.8 [9.7] days) and Medicaid (8.9 [11.7] days; SMD, 0.16). The overall mortality rate of all included patients was 25 181 (8.2%).

After adjusting for relevant covariates, patients who were uninsured underwent earlier WLST compared with those with private insurance (HR, 1.57; 95% CI, 1.49-1.65) and those covered by Medicaid (HR, 1.53; 95% CI, 1.45-1.62) (Table 2). There was no significant difference in time to WLST between Medicaid and the privately insured (HR, 1.03; 95% CI, 0.98-1.08).

**Table 2.** Associations Between Variables of Interest and Time to Withdrawal of Life-Sustaining Therapy

Included variables	HR (95% CI)	*P* value
Insurance type (vs private)		
Medicaid	1.03 (0.98-1.08)	.24
Self-pay	1.57 (1.49-1.65)	<.001
Self-pay insurance (vs Medicaid)	1.53 (1.45-1.62)	<.001
**Covariates**		
Patient factors		
Age^a^	NA	.22
Female sex (compared with male)	0.98 (0.94-1.03)	.43
Race (compared with White)		
African American or Black	0.69 (0.65-0.73)	<.001
American Indian or Native Alaskan	0.82 (0.73-0.92)	<.001
Asian	0.85 (0.78-0.92)	<.001
Native Hawaiian or Pacific Islander	0.93 (0.65-1.31)	.67
Other^b^	1.18 (1.05-1.32)	.006
Hispanic ethnicity (vs not Hispanic)	0.81 (0.75-0.87)	<.001
Trauma Comorbidity Index	1.65 (1.59-1.72)	<.001
**Injury factors**		
AIS		
Head	1.60 (1.57-1.62)	<.001
Face	0.90 (0.88-0.92)	<.001
Neck	1.09 (1.06-1.11)	<.001
Chest	1.09 (1.07-1.10)	<.001
Abdominal	1.09 (1.08-1.11)	<.001
Spine	1.05 (1.04-1.07)	<.001
UE	0.88 (0.86-0.90)	<.001
LE	1.00 (0.99-1.02)	.79
Coma, presenting GCS <8	1.71 (1.55-1.88)	<.001
Trauma type (vs fall)		
Firearm	2.01 (1.79-2.27)	<.001
MVC	0.74 (0.70-0.79)	<.001
Motorcycle	0.78 (0.73-0.84)	<.001
Pedestrian	0.87 (0.81-0.93)	<.001
Stab	1.04 (0.84-1.27)	.74
Struck	0.90 (0.79-1.02)	.08
Other	1.46 (1.17-1.83)	<.001
Intent (vs unintentional)		
Assault	0.87 (0.77-0.99)	.03
Other	0.77 (0.55-1.09)	.14
Self-inflicted	1.54 (1.36-1.73)	<.001
Undetermined	1.05 (0.86-1.28)	.64
Work-related	0.81 (0.74-0.90)	<.001
Presenting BP, per 10 mm HG systolic	0.97 (0.96-0.97)	<.001
ED GCS motor	0.74 (0.73-0.76)	<.001
Interfacility transfer	0.83 (0.79-0.87)	<.001
Hospital factors		
Teaching hospital	0.82 (0.75-0.89)	<.001
For-profit hospital	0.79 (0.70-0.88)	<.001
Bed size (vs >600)		
≤200	0.94 (0.89-1.00)	.04
201-400	1.01 (0.93-1.10)	.83
401-600	0.97 (0.90-1.05)	.45

Abbreviations: AIS, Abbreviated Injury Score; BP, blood pressure; ED, emergency department; GCS, Glasgow Coma Scale; HR, hazard ratio; LE, lower extremity; MVC, motor vehicle collision; NA, not available; UE, upper extremity.

^a^ Cubic spline.

^b^ Other race is inclusive of patients whose race was unknown, not reported or missing.

With respect to other variables considered in the multivariable model, Asian patients (HR, 0.85; 95% CI, 0.78-0.92), Black patients (HR, 0.69; 95% CI, 0.65-0.73), and Hispanic patients (HR, 0.81; 95% CI, 0.75-0.87) were less likely to undergo WLST compared with non-Hispanic White patients. Firearm (HR, 2.01; 95% CI, 1.79-2.27) and self-inflicted (HR, 1.54; 95% CI, 1.36-1.73) injuries were associated with earlier withdrawal of care. Patients treated at teaching hospitals (HR, 0.82; 95% CI, 0.75-0.89) and for-profit hospitals (HR, 0.79; 95% CI, 0.70-0.88) were less likely to WLST.

Our sensitivity analysis including the subset of patients who survived longer than 48 hours did not meaningfully change our results (eTable in Supplement 1). Our exploration of nonwithdrawal death as a competing risk did not yield results indicating the need to change our analysis model to a cause-specific hazard or subdistribution hazard model (eTable in Supplement 1).

## Discussion

In this retrospective study of trauma patients, we found that patients who are uninsured underwent earlier WLST compared with those with private or Medicaid insurance. This association was robust to sensitivity analyses accounting for delays to obtaining insurance and the competing risk of nonwithdrawal deaths. Our study suggests that a patient’s ability to pay may be associated with a shift in decision-making for WLST, which could indicate that socioeconomic status informs end-of-life care for some patients with critical injuries.

To our knowledge, our study is the first large cohort study to examine the association between insurance status and WLST. Our novel finding that patients who are uninsured are at a higher risk for early WLST builds upon previous studies that associated uninsured status with increased mortality in trauma patients.^15,20,21,22^ These prior works have detailed the potential social factors that may contribute to discrepancies in outcomes between patients who are uninsured and insured. One factor to consider as it related to WLST is the availability and level of involvement of SDMs in the clinical decisions. Patients belonging to socially disadvantaged groups may be less likely to have SDMs who are available, able, or willing to participate in treatment discussions. In this setting, clinicians may be more likely to proceed with earlier WLST in patients with a poor prognosis given the inability to have extensive discussion and family consultation before deciding, which can be time consuming. This represents 1 of the possible contributing social factors as to why patients who are uninsured were more likely to undergo earlier WLST. Our present study also suggests that a contributor to the increased rates of mortality in these prior studies could be from decisions to withdrawal or not proceed with treatment among patients who are uninsured. Previous studies that have shown patients who are critically injured and uninsured are less likely to undergo diagnostic investigations and interventional procedures.^6,7,8^ When presented with decisions about whether or not to proceed with tests, procedures, or care continuation, institutions and/or SDMs may both have concerns with the cost of care and be less likely to pursue extensive measures, resulting in earlier mortality. However, we similarly found higher rates of nonwithdrawal deaths in patients who are uninsured in our cause-specific hazards analysis, demonstrating patients who are uninsured have higher risks of both WLST and death from other causes.

A 2015 study by Osler et al^14^ questioned the legitimacy of the association between insurance status and mortality in trauma patients, stating that previous studies reporting this association lacked confounder control (specifically gunshot mechanism and shock on admission) and did not account for survivor treatment assignment bias in the insurance variable. The survivor treatment assignment bias phenomenon exists due to the patients’ insurance status being considered as a baseline characteristic in studies, when in fact it is recorded at their discharge or death, with patients who are uninsured who live longer being more likely to become insured during their stay than those who die early. After controlling for these issues in their models, the authors found no association between insurance status and mortality. In a sensitivity analysis that mirrored their approach, we found that the association between insurance status and earlier WLST still held true. Moreover, our cause-specific hazards model exploring nonwithdrawal deaths also demonstrated increased mortality among patients who are uninsured, thereby aligning with the findings of the studies criticized by Osler et al.^14,15,20,21,22^ The overall rate of WLST in the present study (4%) was similar to previous studies examining similar populations.^23,24,25^

When comparing those insured by Medicaid with patients who are privately insured, there were minimal differences in rates of WLST. This finding was true despite Medicaid patients appearing most similar to the uninsured group at baseline. This finding would suggest that insurance status itself may be a more important estimator of WLST timing than patient demographics or characteristics. However, it is also important to consider that patients enrolled in Medicaid may have greater support structures or face fewer systematic barriers to obtaining insurance than those who remain uninsured, and these social differences might contribute to discrepancies in WLST decisions. Furthermore, while discrepancies in hospital renumeration may exist between private and Medicaid insurance payouts for equivalent care, this did not result in earlier WLST for Medicaid patients. The potential financial concerns for SDMs of patients who are uninsured considering care continuation are likely not as present for SDMs of patients whose medical costs will be covered by Medicaid. From an institutional and clinician perspective, there may be a larger perceived difference when comparing the financial costs of pursuing heroic measures for an insured patient with an uninsured patient, who is unlikely to pay, than there is when comparing the incremental differences between renumeration amounts in patients that are differently insured.

Secondarily, we analyzed the association between other factors included in our model and time to WLST. We found that Asian, Black, and Hispanic patients were less likely to undergo WLST than non-Hispanic White patients. In an exploratory manner, we also examined the interplay between race and insurance status and found that no significant interaction existed, indicating there may be similar WLST practices within racial groups across different insurance types. Previous studies examining end-of-life care in minority populations within the US have demonstrated increased use of life-support, decreased use of hospice, and an increased desire to be kept alive regardless of severity of illness.^26,27,28,29^ In our study, the decreased likelihood of WLST was countered by an increased likelihood of nonwithdrawal death for these populations, suggesting that mortality may remain similar across races after controlling for injury severity and comorbidities despite differences in end-of-life decision-making. Other studies have offered explanations for why patients from minoritized racial and ethnic groups are less likely to have WLST, including the presence of language barriers between family and the health care team, discrepancies in health literacy, and decreased trust in health care professionals.^30,31,32^ In population or large database studies, race and ethnicity may be a surrogate for how cultural and religious beliefs inform the patient and family wishes during end-of-life care and WLST decision-making.

In 2014, the Patient Protection and Affordable Care Act introduced a federal mandate for personal health insurance with a financial penalty for those who were uninsured without exemption. This individual mandate penalty was repealed in 2019 allowing individuals to remain uninsured without direct disincentive.^33^ A recent US Centers for Disease Control and Prevention publication^34^ estimates that 12.2% of Americans aged 18 to 64 years are uninsured. The groups at highest risk of being uninsured included those of Hispanic ethnicity, those with family incomes less than twice the federal poverty line, and those who lived in non-Medicaid expansion states.^34^ In our study, and as previously documented, patients who are uninsured experience disproportionately high rates of traumatic injury, with the uninsured representing a greater proportion of the trauma population compared with the general population.^35^ Our findings suggest that there is a fundamental problem for equity of care, as the ability to pay may affect decisions to withdraw life sustaining therapy for patients who are uninsured.

### Limitations

This study has limitations. First, as with all retrospective cohort studies, our modeling and risk adjustment are limited to variables that are recorded and accessible within the TQIP database. Thus, we were not able to include personal or family income or religious and spiritual beliefs in our analysis, which we believe may have aided in our ability to further explain our findings. Similarly, controlling for geographic location of the treating hospital may have helped account for some cultural influence on both patients and clinicians as well as state-based influences of Medicaid expansion eligibility,^34,36^ though clustering by institution likely reduced some confounding in this regard. No data for marital status, language ability, or education level were available for inclusion. Second, we included comorbidities in our model to control for prognostic differences; however, the diagnosis of a comorbid condition often depends on the prior use of primary care or the health care system. Patients who are marginalized or uninsured are less likely to seek regular primary care, and thus are less likely to have documented chronic disease diagnoses, such as hypertension.^37^ This phenomenon may partly explain why hypertension and smoking status are often found to be protective in trauma prediction models,^16,38,39^ since they either require prior documentation or a length of stay long enough for their discovery. Our analysis excluded patients who lived less than 48 hours, which likely helped reduce this confounding because it gives time for comorbidity diagnosis and reporting. Third, we did not analyze the types of care initiated prior to determining WLST.

## Conclusions

In this cohort study of US adult trauma patients who were critically injured, patients who were uninsured underwent earlier WLST compared with those with private or Medicaid insurance. Based on our findings, a patient’s ability to pay was likely associated with a shift in decision-making for WLST, suggesting that socioeconomic status affects patient outcomes.

## Notes

Supplement 1. **eTable.** Sensitivity Analyses: Inclusive of Patients Surviving More Than 48 Hours, a Competing Risk Analysis With Cause Specific Hazards and Subdistribution Hazards Model (Fine and Gray)

Supplement 2.
Data Sharing Statement
