# Supplementary material for: Insurance Type and Withdrawal of Life-Sustaining Therapy in Critically Injured Trauma Patients
Source: JAMA Netw Open. 2024 Jul 24;7(7):e2421711. doi: 10.1001/jamanetworkopen.2024.21711 (PMC11270131; doi:10.1001/jamanetworkopen.2024.21711)
Supplement: Supplement 1. — eTable. Sensitivity Analyses: Inclusive of Patients Surviving More Than 48 Hours, a Competing Risk Analysis With Cause Specific Hazards and Subdistribution Hazards Model (Fine and Gray) [file jamanetwopen-e2421711-s001.pdf]

Supplemental Online Content

Hoit G, Wijeysundera DN, Hamd DM, et al. Insurance type and withdrawal of life-sustaining therapy in critically injured trauma patients. *JAMA Netw Open*. 7(7):e2421711. doi:10.1001/jamanetworkopen.2024.21711

eTable. Sensitivity Analyses: Inclusive of Patients Surviving More Than 48 Hours, a Competing Risk Analysis With Cause Specific Hazards and Subdistribution Hazards Model (Fine and Gray)

This supplemental material has been provided by the authors to give readers additional information about their work.

© 2024 Hoit G et al. *JAMA Network Open.*

eTable 1: Sensitivity Analyses: Inclusive of Patients Surviving More Than 48 Hours, a Competing Risk Analysis With Cause Specific Hazards and Subdistribution Hazards Model (Fine and Gray)

	Insurance Type (vs Private)	Hazard ratio	95%CI L	95%CI H	p-value
Survived >48hrs Model	Medicaid	1.04	0.99	1.09	0.13
	Self-Pay	1.54	1.46	1.63	<0.001
CSH Model WLST	Medicaid	1.02	0.98	1.07	0.36
	Self-Pay	1.56	1.49	1.65	<0.001
CSH Model Death	Medicaid	0.92	0.87	0.97	0.001
	Self-Pay	1.85	1.77	1.97	<0.001
SDH Model	Medicaid	1.10	1.04	1.16	0.002
	Self-Pay	1.33	1.25	1.41	<0.001

Abbreviations: 95% CI L=95% confidence interval lower value, 95% CI H=95% confidence interval higher value, hrs=hours, CSH=cause specific hazards, WLST=withdrawal of life-sustaining therapy, SDH=subdistribution hazards

© 2024 Hoit G et al. *JAMA Network Open.*
