# Supplementary material for: Insurance Type and Withdrawal of Life-Sustaining Therapy in Critically Injured Trauma Patients
Source: JAMA Netw Open. 2024 Jul 24;7(7):e2421711. doi: 10.1001/jamanetworkopen.2024.21711 (PMC11270131; doi:10.1001/jamanetworkopen.2024.21711)
Supplement: Supplement 2. — Data Sharing Statement [file jamanetwopen-e2421711-s002.pdf]

Data Sharing Statement

Hoit. Insurance Type and Withdrawal of Life-Sustaining Therapy in Critically Injured Trauma Patients. *JAMA Netw Open*. Published July 24, 2024.
doi:10.1001/jamanetworkopen.2024.21711

Data
Data available: Yes
Data types: Data dictionary, Deidentified participant data
How to access data: Anyone with access to TQIP, email for data dictionary and specific data to graeme.hoit@mail.utoronto.ca
When available: With publication

Supporting Documents
Document types: Statistical/analytic code
How to access documents: email to graeme.hoit@mail.utoronto.ca
When available: With publication

Additional Information
Who can access the data: anyone requesting the data, who has approval for ACS-TQIP data
Types of analyses: for any purpose
Mechanisms of data availability: anyone requesting the data, who has approval for ACS-TQIP data
